# Targeted Dual Intervention-Oriented Drug-Encapsulated (DIODE) Nanoformulations for Improved Treatment of Pancreatic Cancer

**DOI:** 10.3390/cancers12051189

**Published:** 2020-05-08

**Authors:** Vijay Sagar Madamsetty, Krishnendu Pal, Shamit Kumar Dutta, Enfeng Wang, Debabrata Mukhopadhyay

**Affiliations:** Department of Biochemistry and Molecular Biology, Mayo Clinic Florida, 4500 San Pablo Road S, Jacksonville, FL 32224, USA; madamsetty.vijay@mayo.edu (V.S.M.); pal.krishnendu@mayo.edu (K.P.); Dutta.Shamit@mayo.edu (S.K.D.); Wang.Enfeng@mayo.edu (E.W.)

**Keywords:** pancreatic cancer, nanoformulations, liposomes, targeted drug delivery, gemcitabine

## Abstract

Despite recent advancements, effective treatment for pancreatic ductal adenocarcinoma (PDAC) has remained elusive. The overall survival rate in PDAC patients has been dismally low due to resistance to standard therapies. In fact, the failure of monotherapies to provide long-term survival benefits in patients led to ascension of several combination therapies for PDAC treatment. However, these combination therapies provided modest survival improvements while increasing treatment-related adverse side effects. Hence, recent developments in drug delivery methods hold the potential for enhancing therapeutic benefits by offering cocktail drug loading and minimizing chemotherapy-associated side effects. Nanoformulations-aided deliveries of anticancer agents have been a success in recent years. Yet, improving the tumor-targeted delivery of drugs to PDAC remains a major hurdle. In the present paper, we developed several new tumor-targeted dual intervention-oriented drug-encapsulated (DIODE) liposomes. We successfully formulated liposomes loaded with gemcitabine (G), paclitaxel (P), erlotinib (E), XL-184 (c-Met inhibitor, X), and their combinations (GP, GE, and GX) and evaluated their in vitro and in vivo efficacies. Our novel DIODE liposomal formulations improved median survival in comparison with gemcitabine-loaded liposomes or vehicle. Our findings are suggestive of the importance of the targeted delivery for combination therapies in improving pancreatic cancer treatment.

## 1. Introduction

Pancreatic ductal adenocarcinoma (PDAC) remains one of the most lethal malignancies, having a 5-year survival rate of less than 8% [1]. The reasons behind such poor prognosis have been postulated due to late-stage diagnosis and resistance to standard therapies [2,3,4]. Highly avascular phenotype of PDAC and the presence of a strong protective stromal barrier affect drug delivery and immune surveillance [5,6,7,8]. Due to the complex tumor biology, various combination therapies for pancreatic cancer, such as multiple drug combinations or drug and gene therapies, have been developed [9,10]. Combination treatment options help in improving the therapeutic benefit by sensitizing tumors toward anticancer drugs [11,12]. For instance, nab-paclitaxel (PTX) combined with gemcitabine demonstrates a better median overall survival rate than gemcitabine alone [13,14,15]. Similarly, a prior report showed that first-line treatment with erlotinib/gemcitabine was comparable to FOLFIRINOX in achieving a 1-year survival rate in rash-positive metastatic PDAC (mPDAC) patients [16]. The overall survival of patients was significantly prolonged on the gemcitabine plus erlotinib treatment than the individual treatment arms [17]. In addition, recent reports identified that c-Met is a new therapeutic target for pancreatic cancer, and inhibiting c-Met can control pancreatic cancer tumor growth and metastasis [18]. Interestingly, the combination of c-Met inhibitor XL-184 with gemcitabine demonstrated significant tumor growth inhibition and increased overall survival in gemcitabine resistance pancreatic cancer mice models; however, it ultimately failed in phase 1 trial (NCT01663272) due to dose-limiting toxicity [18,19,20].

Most of the current chemotherapeutic agents do not significantly differentiate between cancerous and healthy cells, which limits the drug’s maximum tolerable dose. Nanotechnology has become an attractive, increasingly used methodology for drug delivery, opening up new landscapes in medicine, through the introduction of smart drug-delivery systems and increasing numbers of nanotherapeutics and nanodiagnostics that are being commercialized or have reached the clinical stage. However, the foremost challenge in cancer research is to develop efficient nanoformulations that can deliver drugs effectively to the tumor site. Targeted delivery methodologies for the treatment of cancer have exposed a steep rise over the past few decades [21,22]. Several nanoformulations with ∼100 nm diameters have been widely used to improve the distribution and tumor accumulation of cancer drugs/genes, including PDAC [23,24,25]. Among them, the liposomes are one of the most tested nanocarriers in the clinical trials and several marketed products are based on liposomes, and liposomes facilitate in intracellular-delivery of anticancer drugs and prolongs the retention-time of encapsulated payload in cancer cells [26,27,28]. For instance, liposomal irinotecan approved by the FDA for the treatment of mPDAC in combination with other drugs. Nevertheless, compared to the superfluous of successful preclinical studies, only a few passively targeted nanoformulations have been approved for clinical trial and none of the actively targeted nanoformulations have crossed the next level from clinical trials [29,30]. Hence, the development of actively tumor-targeting nanoformulations for the delivery of clinically validated drugs in PDAC patients remains an unmet clinical need.

In the present study, we developed several tumor-targeted dual intervention-oriented drug-encapsulated (DIODE) liposomal nanoformulations for delivering two drugs simultaneously to the tumor site. Following extensive characterization of these nanoformulations, they were thoroughly evaluated for their in vitro cellular uptake, cellular cytotoxicities, in vivo biodistribution, and tumor growth inhibition efficacy. Intravenous (I.V.) administration of the liposomal nanoformulations containing dual drugs (gemcitabine as one of the combinations) significantly inhibited tumor growth by increasing gemcitabine sensitivity in orthotopic pancreatic xenografts. In summary, the presently described tumor-targeted DIODE liposomal nanoformulations may provide a future treatment option for PDAC. 

## 2. Results

### 2.1. Preparation and Characterization of Liposomes

**Figure 1:**
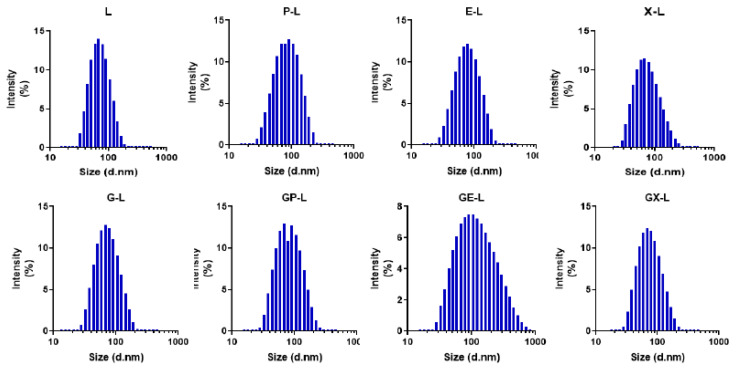
Characterization of liposomes: hydrodynamic diameter histograms of empty (L) or liposomes containing paclitaxel (P-L), erlotinib (E-L), XL-184 (X-L), gemcitabine (G-L) and combination of gemcitabine plus paclitaxel (GP-L), gemcitabine plus erlotinib (GE-L), and gemcitabine plus XL-184 (GX-L) liposomes obtained from dynamic light scattering (DLS) intensity measurements. All the measurements were performed in deionized water at 25 °C.

The single or dual drug-encapsulated tumor-targeted liposomal formulations were prepared by a modified ethanol injection method described previously. The hydrodynamic diameters and zeta potentials of both empty and drug-loaded liposomal formulations were measured by using the dynamic light scattering (DLS) technique. Figure 1 represents the average hydrodynamic diameters (intensity%) of the empty liposomes (L) and drug-loaded liposomes including gemcitabine (G-L), paclitaxel (P-L), erlotinib(E-L), XL-184 (X-L), gemcitabine plus paclitaxel (GP-L), gemcitabine plus erlotinib (GE-L), and gemcitabine plus XL-184 (GX-L). All other liposomal formulations were less than 110 nm of hydrodynamic diameters. Table 1 summarizes all the other physicochemical characteristics, including size, zeta potentials, and polydispersity indices (PDI) values of respective liposomal formulations. The PDI values of all the liposomal formulations were less than 0.3, suggesting the monodisperse nature of the liposomes. The zeta potentials were found to be close to neutral or slightly positive without any significant changes among different liposomes. Moreover, the changes in the hydrodynamic diameter and PDI and zeta potentials of the liposomal formulation were not influenced by the drug encapsulation. Importantly, stability analyses by determining alterations in hydrodynamic diameter in 10% FBS containing RPMI-1640 media (Table S1) demonstrated that all our drug-loaded liposomes showed excellent stability up to 72 h.

### 2.2. Drug-Loading Efficiency and Encapsulation Efficiency

Table 2 summarizes the amount of total lipids and drugs used to formulate the liposomes along with drug-loading efficiency (DLE) and encapsulation efficiency (EE) values for both single and dual drug-loaded liposomes. The drug encapsulation efficiencies for hydrophobic drugs like paclitaxel, erlotinib, and XL-184 were found within the range of 60–80% in both single drug- and dual drug-loaded formulations, whereas the encapsulation efficiency for the hydrophilic drug gemcitabine was less than 26% in both cases. Water-insoluble hydrophobic drugs were naturally arranged within the lipid bilayer of liposomes, whereas water-soluble hydrophilic drugs tended to escape from the lipid bilayer. Consequently, we can see higher entrapment efficiencies for hydrophobic drugs than hydrophilic drugs. The drug-loading efficiencies of both single and dual drugs were less than 5%. No significant variations were observed in both encapsulation efficiencies and drug-loading efficiencies among the single drug-loaded and DIODE liposomes.

### 2.3. In Vitro Cellular Uptake of Rhodamine-PE-Labeled Liposomes in Pancreatic Cancer Cell Lines

**Figure 2:**
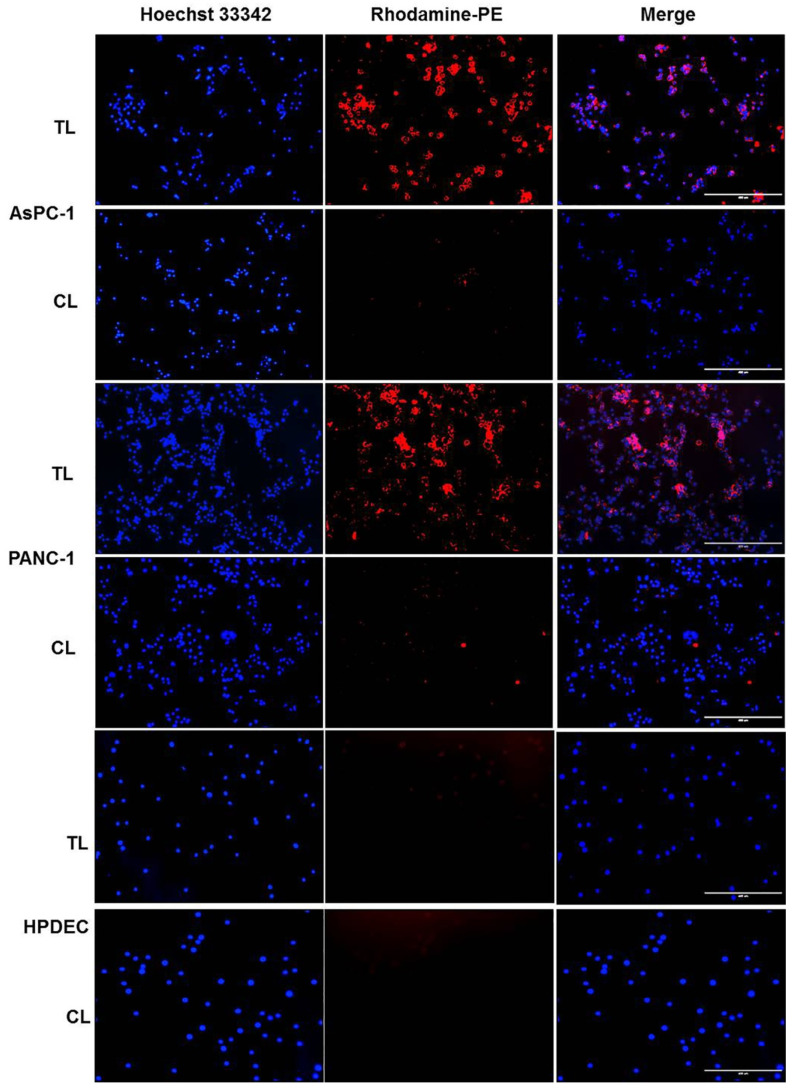
In vitro cellular uptake of Rhodamine-PE-labeled liposomes in pancreatic cancer cell lines. AsPC-1, PANC-1, and HPDEC cells were treated with Rhodamine-PE-labeled TTP-conjugated liposomes (TL) or control liposomes (CL) for 4 h. Nuclei of the cells were counterstained with Hoechst 33342 for the last 30 mins. Finally, cells were washed three times with PBS and images were captured using EVOS fluorescence microscope under the blue and red channels. TL-treated cells showed significantly higher uptake of Rhodamine dye compared to CL-treated cells in AsPC-1 and PANC-1 cells and very minimal uptake in HPDEC cells. Bar length = 400 μm. Abbreviations: TL, tumor targeting peptide-conjugated liposome; CL, control liposome.

Next, we performed in vitro cellular uptake studies using Rhodamine-PE-labeled liposomes to evaluate the targeting efficacy of our newly developed targeted liposomal nanoformulations. Figure 2 shows that the degree of cellular uptake was significantly higher for the Tumor targeting peptide (TTP)-conjugated liposomes (TL) in both AsPC-1 and PANC-1cell lines, whereas minimal cellular uptake was observed for control liposomes (CL). Importantly, we did not observe significant in vitro cellular uptake of both Rhodamine-PE (Rh-PE)-loaded TL and CL liposomes in HPDEC (human pancreatic duct epithelial cell) cells (Figure 2). Additionally, we captured higher magnification images of the cells treated with TL liposomes to better visualize the membrane and cytoplasm localization. Figure S1 summarizes the results. These results suggest the excellent targeting efficiency of TTP-conjugated liposomal formulations.

### 2.4. In Vivo Tumor Uptake Study of NIR-Dye-Loaded Liposomes in Pancreatic Cancer Xenograft

**Figure 3:**
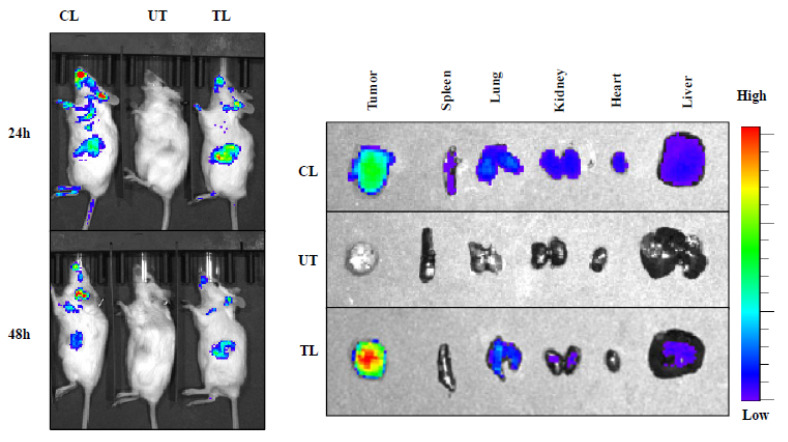
In vivo biodistribution of IR-780-dye-labeled liposomes in orthotopic pancreatic tumor-bearing mice. IVIS (In Vivo Imaging System) imaging showing higher tumor accumulation of IR-780 dye-labeled TTP-conjugated liposomes (TL) compared to control liposomes (CL) at 24 h (upper panel) and 48 h (lower panel) after IV administration into mice bearing AsPC-1 orthotopic tumors in SCID mice. One untreated mouse (UT) was used for background correction. Ex vivo imaging of tumors and major organs harvested at 48 h demonstrated significantly higher tumor uptake of TL compared to CL. Abbreviations: UT, untreated; CL, control liposome; TL, tumor targeting peptide-conjugated liposome.

We further investigated the in vivo tumor targeting efficacy of our newly developed targeted liposomes. Here, we used IR-780-dye-loaded liposomes instead of Rhodamine-PE to avoid the interference of tissue autofluorescence signal usually seen with Rhodamine-PE. NIR dye absorbs and emits in the IR region of the spectrum that is less absorbed by living tissue, and there is no autofluorescence interfering with the signal intensity from mice. We injected IR-780-dye-labeled targeted (TL) and control (CL) liposomes via the intravenous (i.v.) route in mice bearing orthotopic pancreatic tumors developed by injecting AsPC-1 cells. Targeted liposome (TL) showed higher tumor-specific signal compared to control liposome (CL) at 24 and 48 h after i.v. administration (Figure 3). Additionally, the ex vivo imaging of the tumors and major organs also confirmed a high tumor-specific signal for TL than CL (Figure 3). Furthermore, the quantification results of the NIR dye signals of each organ (Figure S2) corroborated with our ex vivo organ imaging results. Collectively, the findings summarized in Figure 2 and Figure 3 are consistent with the supposition that our newly developed tumor-targeting liposomal formulations are efficient in delivering anticancer agents selectively to tumors.

### 2.5. In Vitro Cytotoxicity of Drug-Loaded Liposomes in Pancreatic Cancer Cell Lines

**Figure 4:**
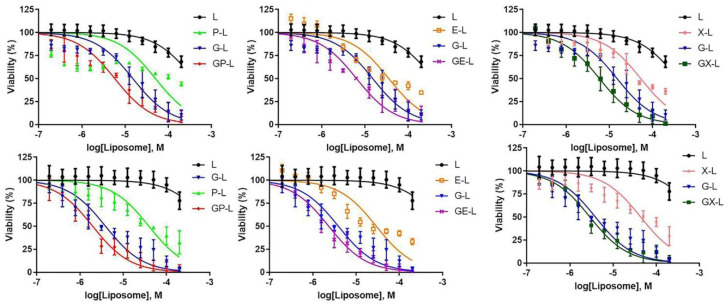
In vitro cellular cytotoxicities of drug-loaded liposomes in pancreatic cancer cell lines. AsPC-1 (upper panel) and PANC-1 (lower panel) cells were treated with various drug-loaded TTP-conjugated liposomes for 72 h. Then, cell viability was determined with cell titer glow assay. Dual drug-loaded liposomes showed a higher reduction in cell viability compared to single drug-loaded liposomes of each combination in all cell lines. Each data point represents the quadruplet results obtained from a single experiment. Abbreviations: L-liposome only; P-L represents liposomal paclitaxel; E-L represents liposomal erlotinib, X-L represents liposomal XL-184; G-L represents liposomal gemcitabine; GP-L represents liposome loaded with both gemcitabine and paclitaxel; GE-L represents liposome loaded with both gemcitabine and erlotinib, GX-L represents liposome loaded with both gemcitabine and XL-184.

After witnessing promising results from the uptake studies, we performed CellTiter-Glo^®^ 2.0 Assay in pancreatic cancer cell lines with a view to examine the cytotoxicity profiles of various drug-loaded liposomal formulations. As our targeted liposomes (TL) showed considerably higher in vitro cellular uptake and in vivo tumor targeting compared to control liposomes (CL), we used TL for all further efficacy studies. The DIODE liposomes showed better cytotoxicity than single drug or empty liposomes in both AsPC-1 and PANC-1 cell lines (Figure 4). Table S2 summarizes the calculated IC-50 values of respective drugs.

### 2.6. In Vivo Efficacy of Drug-Loaded Liposomes in Pancreatic Cancer Xenograft

Subsequently, to further evaluate the in vivo efficacy of our newly developed DIODE liposomal nanoformulations, we utilized a single mouse trial strategy, which is currently being used by various research groups and Charles River Laboratories for PDX avatar models. In this approach, the longitudinal tumor growth was measured for a single mouse per treatment arm to identify the best treatment regimen cost effectively and reliably. To be more clinically relevant, we also started the treatment when the tumors became palpable. We have administered drugs twice a week for 3 weeks via i.v. route with significantly lower doses of each drug than reported in the literature [31,32]. Table S3 summarizes the doses of each drug used in our studies. All DIODE liposomal formulations demonstrated significant inhibition of tumor growth in comparison with their respective single-drug formulations, vehicle, and untreated control in the AsPC-1 tumor model (Figure S3A,B). Moreover, among the DIODE liposomal formulations, gemcitabine plus XL-184 containing liposomes (GX-L) exerted the most inhibitory effect on tumor growth.

We also performed a similar experiment in another tumor model developed using PANC-1 cells. Figure S4A,B summarize the results for tumor volume and tumor weight, respectively. Since PANC-1 tumors grow slowly than highly aggressive AsPC-1 tumors, we have started treatment after 4 weeks of cell implantation in this experiment. We found more or less similar results in this model as well, GX-L being the best among DIODE formulations. 

**Figure 5:**
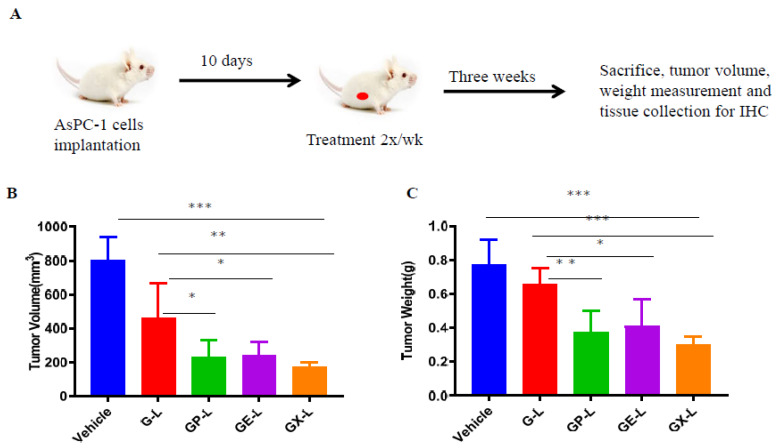
In vivo therapeutic efficacy of drug-loaded liposomes in an orthotopic pancreatic tumor model. Briefly, 1 × 10^6^ AsPC-1 cells were orthotopically injected into the head of the pancreases of 6–8 weeks old female mice. After 10 days of cells implantation, mice were imaged by using IVIS and randomized into five groups (*n* = 5 in each group). Then, mice were treated with indicated groups 2×/week for 3 weeks. (**A**) The graphical representation of in vivo experimental plan. After the end of the treatment, mice were sacrificed, and endpoint tumor volume (**B**) and tumor weight (**C**) were measured. *** denotes *p* < 0.001 compared to control; ** denotes *p* < 0.01 compared to G-L group; and * denotes *p* < 0.05 compared to G-L group. (Abbreviations: G-L represents liposomal gemcitabine; GP-L represents liposome loaded with both gemcitabine and paclitaxel; GE-L represents liposome loaded with both gemcitabine and erlotinib, GX-L represents liposome loaded with both gemcitabine and XL-184).

**Figure 6:**
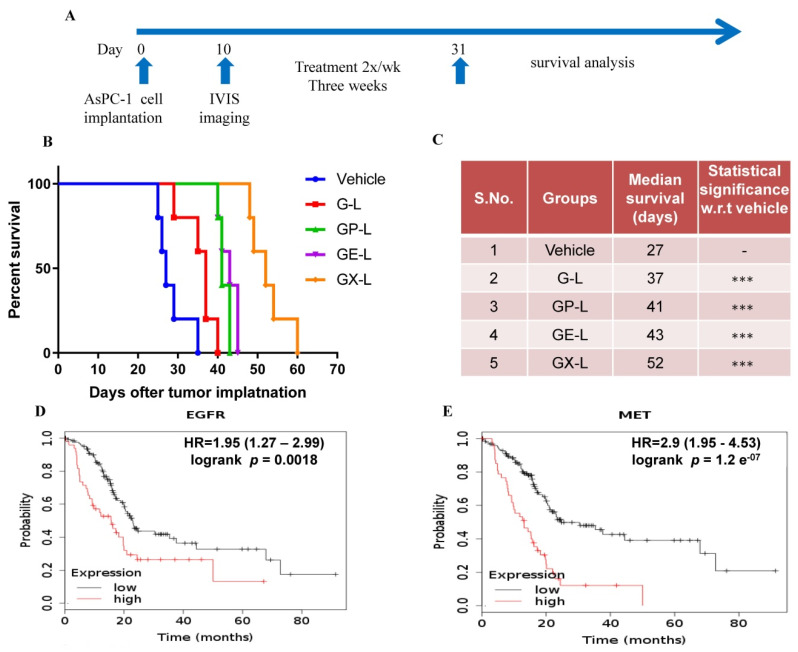
In vivo survival improvement of dual drug-loaded liposomes in an orthotopic pancreatic tumor model. After 10 days of cell implantation, mice were imaged by using IVIS and randomized into five groups (*n* = 5 in each group). Then, mice were treated with indicated groups 2×/week for 3 weeks. After the end of the treatment, mice were observed, and IACUC endpoints in the AsPC-1 tumor model for survival analysis were noted. (**A**) Represents the experimental plan of survival study and (**B**) represents median survival study graph developed using GraphPad software. (**C**) Improved survival days with indicated treatment groups along with statistical significance with respect to the vehicle or G-L Kaplan–Meier survival plots for both EGFR (**D**) and MET (**E**) were obtained from the analysis of the Kaplan–Meier-plotter [Pan-cancer RNA-seq] database. *** denotes *p* < 0.001 compared to control. (Abbreviations: G-L represents liposomal gemcitabine; GP-L represents liposome loaded with both gemcitabine and paclitaxel; GE-L represents liposome loaded with both gemcitabine and erlotinib, GX-L represents liposome loaded with both gemcitabine and XL-184).

To validate our results obtained from single mice experiments, we performed a validation experiment in cohorts of 5 mice in each group using DIODE liposomal formulations. We also included only gemcitabine-loaded liposomes (G-L) in our validation study to compare the benefits of the DIODE liposomal formulations over using G-L. Prior to starting the treatment, we confirmed that each group had more or less equal tumor burden by using bioluminescence imaging of mice with Xenogen IVIS machine (Figure S5A) followed by quantification (Figure S5B). Figure 5A describes our in vivo experimental plan for the therapeutic evaluation study. We observed similar inhibition in tumor volume (Figure 5B) and tumor weight (Figure 5C) that we obtained from single mice experiments. Tumor growth was significantly inhibited in all the treatment groups in comparison with the control group. Tumor volumes in GX-L-treated group (172 ± 30 mm^3^) were significantly smaller in comparison with vehicle (802 ± 134 mm^3^) or G-L (460 ± 206 mm^3^) or GP-L (229 ± 101 mm^3^) and GE-L (239 ± 80 mm^3^) groups (Figure 5B). Similar patterns of results were observed from tumor weight measurements (Figure 5C). The endpoint mice body weights and the observation that mice seemed to be healthier in all liposomal formulations treated groups indicated the nontoxicity effect of our nanoformulations (Figure S6). Another set of experiment was performed to estimate the median survival enhancement of various treatment groups. Figure 6A is a schematic representation of our in vivo experimental plan for survival analysis. GX-L-treated group showed a significant increase (52 days), 190% in median survival in comparison with the control group (27 days) (Figure 6B,C). Other treatment groups, including G-L (36 days), GP-L (41 days), and GE-L (43 days), also showed significantly increased median survival in comparison with the control group (Figure 6B,C). Furthermore, Kaplan–Meier survival plots for both EGFR and MET suggest that PDAC patients with higher expression of EGFR or c-Met exhibit worse survival than patients with lower expressions (Figure 6D,E). We believe that our DIODE liposomal formulations may be beneficial for those patients; however, rigorous validation studies are required before that can happen.

**Figure 7:**
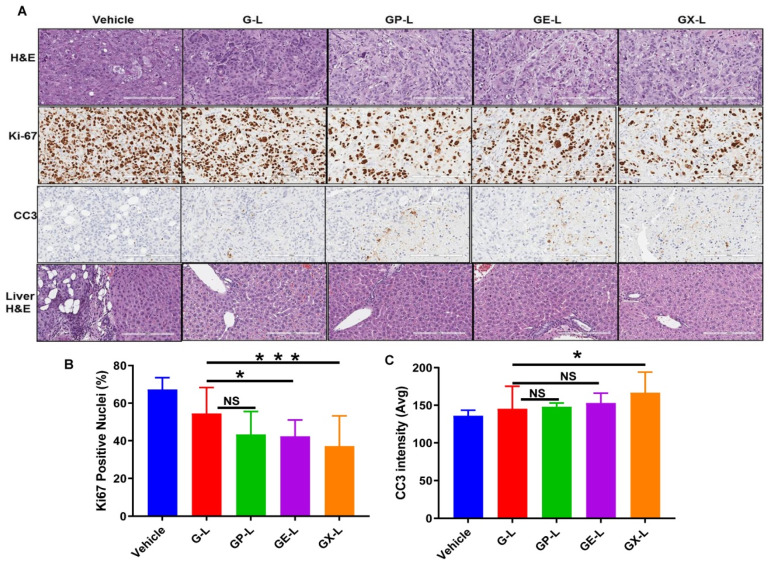
H&E, Ki67, and CC3 staining of tumor sections and liver H&E obtained from respective treatment groups. (**A**) Representative images of the H&E- and Ki67-stained tumor sections from different treatment groups displayed the comparatively higher antiproliferative activity of dual drug-loaded liposomes than gemcitabine-loaded liposomes and untreated. Quantification of Ki67-positive nuclei (**B**) and average CC3 positive intensity (avg) (**C**) of tumor sections. * and *** denotes *p* < 0.05 and *p* < 0.001 compared to G-L, respectively.

To assess the tissue level antiproliferative effects of different treatment groups, tumor sections were stained for antigen Ki-67 protein. The DIODE liposomal formulations GP-L, GE-L, and GX-L, showed less Ki-67 staining compared to G-L or vehicle (Figure 7A, second row). Additionally, the quantification results for Ki67-positive nuclei (Figure 7B) corroborated with our histology results. Furthermore, to evaluate the treatment-induced apoptosis in the tumor tissues, sections were also stained for cleaved caspase 3. The tumor tissues from mice treated with DIODE liposomal formulations (GP-L, GE-L, and GX-L) (Figure 7A, third row) displayed visibly more apoptosis than gemcitabine-loaded (G-L) liposomal formulations or vehicle-treated mice tumor tissues. Similarly, the CC3 quantification data (Figure 7C) was in close agreement with the histology data (Figure 7A, third row). Less nuclear (with hematoxylin and eosin, H&E, Figure 7A, first row) staining in all the treatment group tumor sections than gemcitabine liposome or vehicle-treated liposome indicates more tumor necrosis that happened due to dual drug treatment. Importantly, a substantial reduction of liver micrometastasis was observed in all treatment groups, including G-L, GP-L, GE-L, and GX-L, than vehicle-treated liposome as recognized by H&E-stained liver tissue sections (Figure 7, last row). Finally, Figure S7 denotes that there is no abnormal side effect with our formulations in major organs like lung, kidney, heart, and spleen. In summary, all our findings strongly suggest that our formulations have strong potential in impeding tumor growth and improving the median survival of pancreatic cancer patients with minimal chemotherapy-associated side effects.

## 3. Discussion

Pancreatic ductal adenocarcinoma (PDAC) is one of the lethal causes of cancer-related demise in the United States (US), with less than 8% of 5-year overall survival rate [1]. PDAC accounts for about 3% of all cancers in the US and about 7% of all cancer deaths [33]. It is estimated that in 2020, about 57,600 people will be newly diagnosed, and about 47,050 people will die due to PDAC in the US [34]. These drastic records highlight the urgency of developing novel therapeutic regimens for a better patient outcome. Though gemcitabine (GEM) is the standard of care monotherapy for PDAC, drug resistance developed by tumor cells limits its effectiveness [35]. Numerous research and clinical investigations have documented that coadministration of other drugs along with GEM led to superior therapeutic benefits [36,37,38]. For example, Abraxane plus gemcitabine is currently being used for the treatment of PDAC [14]. However, repeated administrations of such combination therapies result in severe systemic toxicities [14]. Besides, the clinical applications of many anticancer drugs have been hindered due to their limited water solubility and poor pharmacokinetics. Therefore, developing new methodologies to deliver such a combination of therapeutics more selectively to the tumor site is urgently needed. Nanoparticle-mediated delivery systems may be beneficial in this regard by offering multiple-drug-loading capacity, upsurge circulation stability, tumor-specific delivery, and minimal chemotherapy-associated adverse side effects [39,40]. Among them, liposomes can play a vital role in resolving the issues, i.e., off-target properties of anticancer drugs by improving the pharmacological parameters and pharmacokinetic profile.

Hence, in the present study, we have developed DIODE liposomal nanoformulations to find effective treatment options for PDAC. Here, we used the combination of gemcitabine with paclitaxel, erlotinib, and XL-184 to make DIODE liposomal nanoformulations. We evaluated their physical characteristic properties like size, PDI, zeta potentials, and drug-loading efficiencies. All the formulations demonstrated excellent characteristic features that are important for clinical use (Table 1). In the present study, we used DSPE-PEG to develop nice stealth liposomes since it is well documented that PEG inhibits nonspecific protein interaction on the surface of the liposomes and increase their circulation time [41,42,43]. Hydrophobic drugs showed high loading efficacies (60–80%) than hydrophilic drugs (~20%), which is consistent with previous data published by several groups (Table 2) [44,45]. Next, we tested the in vivo tumor targeting efficacy of our novel liposomal nanoformulations, and as expected, we observed higher signal intensity in the case of NIR-dye-loaded targeted liposomes (TL) than control liposomes (CL) (Figure 3). Therefore, we used TL for all further efficacy experiments. In cell viability experiments, as usual, we observed that dual drug-loaded DIODE liposomal nanoformulations showed higher cytotoxicities than corresponding individual drug liposomal formulations.

It is to be noted that, several combinations of the drugs selected for our study have been previously tested for their antitumor efficacy in their parent forms or via nanocarriers in some cases in various cancers including PDAC. For example, a recent report suggested that median progression-free survival (PFS) and overall survival (OS) were improved with Nab-paclitaxel plus gemcitabine treatment in both locally advanced and metastasized PDAC, but hematologic toxicity was observed and dose reductions were performed in most of the patients [13,14]. Erlotinib, which acts as an epidermal growth factor receptor (EGFR) tyrosine kinase inhibitor (TKI) and blocks its downstream signaling cascade to modulate cancer proliferation, differentiation, apoptosis, invasion, and metastasis, has been shown to increase the efficacy of gemcitabine towards radiation or recurrent PDAC tumor growth in mice [46,47,48,49,50]. Despite modest survival benefits observed from gemcitabine and erlotinib in clinical studies, combination treatment of gemcitabine and erlotinib was also approved for use in the advanced disease of PDAC, which prolongs 1-year survival by only 23% [17]. However, their usage in the clinic is limited due to dose-limiting toxicity (DLT). Similarly, some research studies witnessed that inhibiting c-Met impedes PDAC tumor growth and metastasis. The c-Met inhibitor XL-184, in combination with gemcitabine, demonstrated significant tumor growth inhibition and improving overall survival in gemcitabine-resistant pancreatic cancer mice models [18,19]. Notably, both EGFR and c-Met play an important role in drug resistance mechanisms and both are highly overexpressed in many cancer types including PDAC [51,52,53]. 

Furthermore, several research groups have also tried nanocarrier-mediated codelivery for some of the above combinations to improve their efficacy. For example, Nel’s group demonstrated that gemcitabine plus paclitaxel coloaded mesoporous silica nanoparticles (PTX/GEM-loaded LB-MSN) exerted a potential synergistic effect in tumor growth inhibition of pancreatic cancer than only GEM-loaded LB-MSN or gemcitabine plus Abraxane [31]. Another recent study found that gemcitabine plus paclitaxel-loaded liposomes significantly induced apoptosis than only gemcitabine-loaded liposomes in pancreatic cancer cells in vitro [54]. Recently, researchers also developed gemcitabine and PTX coloaded nanoparticles, including liposomes and PLGA nanoparticles for effective cancer therapy [55,56,57]. Several other combinations of drug-based nanoparticle treatments along with gemcitabine were discussed in the recently published review article [37]. Erlotinib-loaded albumin nanoparticles demonstrated their cytotoxic effects against ASPC-1 and PANC-1 cell lines in in vitro settings [58]. Some other groups also established nanocarriers coloaded with erlotinib and doxorubicin or quercetin, etc. for the treatment of cancer [59]. Similarly, XL-184-loaded PEGylated micelles were developed and characterized by Forrest group followed by cytotoxicity studies in vitro [60]. Recently, the Harvard research team developed benzoporphyrin (photoactivable) and XL-184 coloaded nanoliposomes for reducing systemic drug exposure and associated toxicities by a controlled drug release mechanism. The results demonstrated better tumor growth inhibition and suppressed metastatic escape in an orthotopic pancreatic tumor model [32]. Taken together, there is a strong rationale behind the selection of combination drugs used in our study. However, till date, there are no reports of nanoparticles mediated codelivery of gemcitabine with either erlotinib or XL-184 for the treatment of PDAC. 

In the present study, we for the first time, evaluated and compared the above combination therapies in one in vivo system to find the best combination strategy. We evaluated all our DIODE liposomal nanoformulations and their respective individual liposomes in a single mouse trial (SMT) using two different in vivo PDAC xenograft models and validated the best results in cohorts of 5 mice in one of them. Similar to previous reports discussed above, all our newly developed DIODE liposomal nanoformulations showed better antitumor activity than their respective single drug-loaded liposomes in both SMT experiments. Similarly, all DIODE formulations showed better efficacy than gemcitabine-loaded liposomes in the validation experiment. Among all the DIODE liposomal nanoformulations, GX-L showed superior antitumor activity. A decrease in proliferation (as evident from Ki67 staining) and an increase in apoptosis (CC3 staining) support the observed results. Furthermore, all these new formulations inhibited liver metastasis effectively in comparison with the vehicle-treated group; however, we did not observe significant differences among those treatment groups (Figure 6). Nonetheless, survival study experiments of these DIODE nanoformulations demonstrated that all the DIODE liposomal nanoformulations showed significantly improved median survival than G-L and vehicle, suggesting a better therapeutic response of our DIODE formulations. As expected, GX-L showed the highest median survival benefit among all. In addition, our nanoformulations do not show any severe systemic toxicity and noticeable alterations in endpoint mice body weight suggesting that they are well tolerated. This is one of the crucial characteristics for better patient outcomes in addition to the antitumor effect. Importantly, we have tested the in vivo therapeutic efficacy of one liposomal formulation (X-L) in combination with gemcitabine in C57BL6J mice (nonimmune compromised mice) tumor model developed by inculcating KPC cell lines. Figure S8A,B summarize the results for the final tumor volume and the final tumor weights of the respective groups. The treatment of X-L significantly improved the tumor growth inhibition of gemcitabine in comparison with only gemcitabine treatment group (Figure S8). The results demonstrated that our formulations work well in nonimmune compromised tumor models, which are closely related to clinical settings. However, in-depth toxicity studies and therapeutic efficacy studies are needed to be evaluated for the best formulation before going to the next level.

## 4. Materials and Methods

### 4.1. Materials

1,2-Dioleoyl-sn-glycero-3-PC (DOPC) was purchased from Cayman Chemicals, Ann Arbor, MI, USA and 1,2-distearoyl-sn-glycero-3-phosphoethanolamine-N-[methoxy(polyethylene glycol)] (DSPE-(PEG)2000-OMe) was purchased from Nanosoft Polymers, Winston-Salem, NC, USA. Cholesterol and absolute ethanol were obtained from Sigma-Aldrich, St. Louis, MO, USA. Gemcitabine, paclitaxel, XL-184, and erlotinib were procured from LC Laboratories, Woburn, MA, USA. DMEM, RPMI media, and Pen-Strep solution were purchased from Gibco, Gaithersburg, MD, USA. Fetal bovine serum was purchased from Gemini Bioproducts, West Sacramento, CA, USA. Rabbit polyclonal Ki-67 and cleaved caspase-3 antibodies were purchased from Abcam, Cambridge, MA, USA. Milli Q water was used for all the experiments.

### 4.2. Cell Culture

AsPC-1 cells were maintained in RPMI 1640 medium, PANC-1 cells were maintained in Dulbecco’s Modified Eagle Medium, and both cells medium supplemented with 10% fetal bovine serum, 1% anti-anti; Gibco, and 0.02% Plasmocin (InvivoGen, San Diego, CA, USA) at 37 °C in a humidified incubator with 5% CO_2_. HPDEC cells were grown in keratinocytes/serum-free medium supplemented with 0.2 ng of EGF (Gaithersburg, MD, USA) and 30 μg/mL bovine pituitary extract at 37 °C in a humidified incubator with 5% CO_2_.

### 4.3. Preparation Drug/Drugs-Loaded Liposomes

TTP (a tumor-targeting-peptide with a proprietary sequence)-conjugated lipopeptide was synthesized using the Fmoc-strategy-based solid-phase peptide synthesis method described in our recent publication [61]. All the liposomes were prepared by the ethanol injection method with some modifications [62]. Briefly, the dried film of required amounts of DOPC, cholesterol, DSPE-(PEG)2000-OMe, and TTP-conjugated lipopeptide was redissolved in absolute ethanol and warmed in a 65 °C for 5 min before making liposomes. Then, this ethanolic solution of lipids was gently added to preheated Milli-Q water under stirring condition. Stirring was continued for 1 min at room temperature. Finally, ethanol was evaporated employing a rotary evaporator, and volume was made up with milli-Q water. For making drug-loaded liposomes, the required amount of ethanolic solution containing respective single or double drugs was mixed with dried lipids film and the same procedure was followed as described above. Amicon Ultra centrifugal filters with a cut off size of 3 k were used to remove entrapped drugs.

### 4.4. Physical Characterization and Drug Loading of Liposomes

The mean sizes and zeta potentials of various single or dual drug-loaded liposomes were measured by using Malvern Zetasizer Nano ZS (Malvern instruments inc, Westborough, MA, USA) at 25 °C. All the samples were diluted with water for analysis, and the analysis was performed in triplicate. The entrapment efficiencies of all the drugs used were determined by high-performance liquid chromatography (HPLC) (Jasco, Easton, MD, USA). Liposome-entrapment efficiency was measured by determining the amount of unentrapped drugs. Briefly, the encapsulated drug (E_drug_) amount was calculated by subtracting the amount of unentrapped drugs (U_drug_) from the total drug (T_drug_) amount. The drug-entrapment efficiency (EE) was expressed as the percentage of the entrapped amount (E_drug_) to the total amount (T_drug_). The drug-loading efficiency (DLE) was calculated as the percentage of the encapsulated amount (E_drug_) to the total lipid amount (T_lipid_).

### 4.5. Cellular Uptake Assay of Rhodamine-PE-Labeled Liposomes

AsPC-1 cells, PANC-1, and HPDEC cells were seeded in 96-well plates at a density of 5 × 10^3^ cells per well, respectively, and incubated for 24 h. Rhodamine-PE-labeled targeted liposomes (TL) and control liposomes (CL) were added into wells and incubated for 4 h. Cells were stained with 1 μg/mL Hoechst for the last 30 min of incubation. Finally, cells were washed three times with PBS and immediately imaged with EVOS FL Auto fluorescent microscope under blue and red channels. 

### 4.6. In Vitro Cytotoxicity Assay

AsPC-1 cells and PANC-1 cells were plated in 384-well plates at a density of 2 × 10^3^ cells and incubated for 24 h. Cells in 96-well plates were incubated with different drug-loaded formulations (at final liposomes concentrations of 200 μM to 200 nm) and again incubated for 72 h. After 72 h, cell viabilities were assayed using CellTiter-Glo® 2.0 Assay (Promega) as per the manufacturer’s protocol. Briefly, 24 μL of reagent was added to each well. The plates were incubated for 10 min at RT, and luminescence signals were measured using SpectraMax i3x. Percentage viability was calculated as follows: Viability (%) = 100 × (L_Treated_ − L_Blank_)/(L_Untreated_ − L_Blank_).

### 4.7. Animal Experiments

Six-to eight-week-old female SCID mice and C57BL/6J mice (weighing ∼22−25 g) were obtained from in-house breeding and monitored under standard housing conditions in the institutional animal facilities.

### 4.8. Ethics Committee Approval

All animal studies described in this manuscript were performed under the guidelines of NIH, USDA (code: 41-R-0006) and AAALAC (Ethic code: 000860, 03/15/2016) and protocols approved by the Mayo Clinic Institutional Animal Care and Use Committee (IACUC), Florida, USA. All the research was performed as per the Guide for the Care and Use of Laboratory Animals, 8th edition.

### 4.9. In Vivo Tumor-Targeting Evaluation

To evaluate the tumor-targeting efficiency of our targeted liposomes, the tumor-bearing models were established by orthotopic inoculation of 1 × 10^6^ AsPC-1 cells. After 3 weeks, when the tumors became palpable, both targeted and control liposomes loaded with NIR dye were intravenously injected. The mice were then anesthetized and imaged using IVIS (Caliper, Hopkinton, MA, USA) at 24 and 48 h after administration. To further confirm the increased targeting efficiency, mice were sacrificed, and major organs were collected and imaged ex vivo. The fluorescence signals of each organ from ex vivo results were quantified by subtracting the fluorescence signals of a respective organ obtained from untreated mice.

### 4.10. In Vivo Antitumor Efficacy and Survival Study

A single mouse trial (SMT) or Avatar model was used to evaluate the in vivo tumor regression efficacy of the drug-loaded liposomes in AsPC-1 and PANC-1 xenografts, as described previously [61,63]. Briefly, 1 × 10^6^ GFP-Luci-AsPC-1 or GFP-Luci-PANC-1 cells were orthotopically injected into the head of the pancreas of female SCID mice. After 10 days and 4 weeks of AsPC-1 and PANC-1 tumor cell implantation, respectively, mice were imaged using IVIS by luminance imaging. Mice having similar luciferase signal were randomly selected for treatments, i.e., empty liposomes (L) and gemcitabine (G-L)-, paclitaxel (P-L)-, erlotinib (E-L)-, XL-184 (X-L)-, gemcitabine plus paclitaxel (GP-L)-, gemcitabine plus erlotinib (GE-L)-, and gemcitabine plus XL-184 (GX-L)-loaded liposomes. All the treatments were done via tail veins at a dose of 100 uL of each liposome twice a week for 3 weeks. Two days after the last treatment, the mice were euthanized, and tumors, hearts, livers, spleens, lungs, and kidneys of mice were collected. Tumors were weighed and also measured by using calipers, and tumor volumes were calculated using the formula ½ (a × b^2^) where a and b are the longest and shortest diameters, respectively. To validate the results obtained from the SMT, we repeated the experiment in cohorts of 5 mice per group with the most effective treatment group and vehicle control in AsPC-1 tumor-bearing mice. Finally, in order to estimate median survival enhancement, we also performed a survival study experiment in AsPC-1 tumor-bearing mice. After completion of the abovementioned treatments, we noted the IACUC endpoint of the mice as the survival termination date. Kaplan–Meier survival plots for both EGFR and MET were obtained from the analysis of the Kaplan–Meier-plotter [Pan-cancer RNA-seq] database [64].

### 4.11. Immunohistochemistry

Tumors and organs were fixed in 10% formalin buffer at room temperature for 24 h before embedding in paraffin sectioning. Sections were deparaffinized and exposed to hematoxylin and eosin (H&E), cleaved caspase 3 (CC3), and Ki67 immunochemistry according to the manufacturer’s instructions (DAB 150; Millipore, Burlington, MA, USA). Stable diaminobenzidine and hematoxylin were used as the chromogen substrate and the counterstain, respectively. Photographs of cross-sections were digitalized with the Aperio AT2 slide scanner (Leica, Buffalo Grove, IL, USA). Images were analyzed using ImageScope software (Leica).

### 4.12. Statistical Analysis

Data were expressed as mean ± SEM and comparison between groups was performed by repeated-measures analysis of unpaired Student *t* test using GraphPad Prism software (GraphPad Software Inc., San Diego, CA, USA.). *p* < 0.05 was considered statistically significant.

## 5. Conclusions

In summary, we have developed new biodegradable targeted DIODE liposomal nanoformulations for the treatment of pancreatic cancer. The prepared liposomal formulations were of nanosized range (50–101 nm) with slight positive zeta potential. They showed excellent colloidal stability in 10% FBS containing RPMI-1640 media up to 72 h. These DIODE nanocarriers showed improved therapeutic benefit in both in vitro and in vivo settings and offer a promising new approach for improving drug delivery for the treatment of solid tumors such as PDAC. Taken together, the results obtained from this study are of high clinical significance and provide justification for future clinical trials with these DIODE formulations to potentiate standard chemotherapy regimens and improve survival.

## Figures and Tables

**Table 1:** Hydrodynamic diameter, polydispersity index (PDI), and zeta potentials of respective liposomes.

LIPOSOME	SIZE (nm)	PDI	ZETA (mV)
L	69.15 ± 0.98	0.170 ± 0.007	23.5 ± 4.08
P-L	67.92 ± 0.65	0.184 ± 0.009	16.3 ± 2.7
E-L	68.60 ± 0.79	0.172 ± 0.003	15.9 ± 0.95
X-L	71.06 ± 0.38	0.145 ± 0.003	17.4 ± 2.56
G-L	69.99 ± 0.69	0.151 ± 0.011	10.2 ± 2.45
GP-L	68.85 ± 0.76	0.157 ± 0.001	13.6 ± 0.60
GE-L	101.12 ± 1.47	0.321 ± 0.033	11.8 ± 1.10
GX-L	69.82 ± 1.004	0.168 ± 0.010	17.6 ± 1.73

**Table 2:** Total lipid weight, initial drug amount added, drug-loading efficiency (DLE), and encapsulation efficiency (EE) of single or dual drug-loaded liposomal formulations.

Liposome	Total Lipid (mg/mL)	Initial Drug Added (mg/mL)				DLE (%)	EE (%)
		G	P	E	X		
**L**	5.487	–	–	–	–	–	–
**P-L**	5.487	–	0.1			1.5 ± 0.02	81.1 ± 1.5
**E-L**	5.487	–	–	0.2	–	2.6 ± 0.12	70.7 ± 3.9
**X-L**	5.487	–	–	–	0.150	1.82± 0.07	66.5 ± 2.9
**G-L**	5.487	1	–	–		3.3 ± 0.39	18.1 ± 2.1
**GP-L**	5.487	1	0.1			4.5 ± 0.15(G); 1.45 ± 0.07(P)	24.6 ± 0.8(G); 79 ± 3.8(P)
**GE-L**	5.487	1	–	0.2		3.1 ± 0.32(G); 2.8 ± 0.08(E)	16.9 ± 1.7(G); 77.3 ± 2.3(E)
**GX-L**	5.487	1	–	–	0.150	4.8 ± 0.06(G); 2 ± 0.06(X)	26.6 ± 0.37 (G); 75.4 ± 2.2(X)

Abbreviations: G-gemcitabine; P-paclitaxel; E-erlotinib; X-XL-184; DLE, drug-loading efficiency; EE, encapsulation efficiency.

## Supplementary Materials

The following are available online at https://www.mdpi.com/2072-6694/12/5/1189/s1. Figure S1: in vitro cellular uptake of Rhodamine-PE-labeled liposomes in pancreatic cancer cell lines. AsPC-1, PANC-1, and HPDEC cells were treated with Rhodamine-PE-labeled TTP-conjugated liposomes (TL) for 4 h. Nuclei of the cells were counterstained with Hoechst 33342 for the last 30 min. Finally, cells were washed three times with PBS, and images were captured using the EVOS fluorescence microscope under the blue and red channels in 40× magnification. Bar length = 100 μm; Figure S2: quantification of fluorescence signals obtained from ex vivo organ distribution of IR-780-dye-labeled liposomes in orthotopic pancreatic tumor-bearing mice. Higher fluorescence signals were observed in tumors treated with IR-780-dye-labeled-TL-liposomes than tumors treated with IR-780-dye-labeled CL-liposomes after 48 h IV administration into mice bearing AsPC-1 orthotopic tumors in SCID mice. Abbreviations: CL, control liposome; TL, tumor targeting peptide-conjugated liposome; Figure S3: in vivo single mouse trial of drug-loaded liposomes in AsPC-1 xenograft. Briefly, 1 × 10^6^ AsPC-1 cells were orthotopically injected into the head of the pancreases of 8 weeks old female SCID mice. After 10 days, mice were treated with drug-loaded liposomes (one mouse per treatment group) 2×/week for 3 weeks. Then, mice were sacrificed, tumors were harvested, and tumor volumes (**A**) and tumor weights (**B**) were measured. In all cases, dual drug-loaded liposomes demonstrated higher inhibition compared to single drug-loaded liposomes. (Abbreviations: UT-untreated control; L-liposome only; G-L represents liposomal gemcitabine; P-L represents liposomal paclitaxel; E-L represents liposomal erlotinib, X-L represents liposomal XL-184, GP-L represents liposome loaded with both gemcitabine and paclitaxel; GE-L represents liposome loaded with both gemcitabine and erlotinib, GX-L represents liposome loaded with both gemcitabine and XL-184); Figure S4: in vivo single mouse trial of drug-loaded liposomes in PANC-1 xenograft. Briefly, 1 × 10^6^ GFP-luciferase-labeled PANC-1 cells were orthotopically injected into the head of the pancreases of SCID mice. After 4 weeks of cell implantation, mice were treated with drug-loaded liposomes (one mouse per treatment group) 2×/week for 3 weeks. Then, mice were sacrificed, tumors were harvested, and tumor volumes (**A**) and tumor weights (**B**) were measured. In all cases, dual drug loaded-liposomes demonstrated higher inhibition compared to single drug-loaded liposomes. (Abbreviations: UT-untreated control; L-liposome only; G-L represents liposomal gemcitabine; P-L represents liposomal paclitaxel; E-L represents liposomal erlotinib, X-L represents liposomal XL-184, GP-L represents liposome loaded with both gemcitabine and paclitaxel; GE-L represents liposome loaded with both gemcitabine and erlotinib, GX-L represents liposome loaded with both gemcitabine and XL-184); Figure S5. initial tumor bioluminescence signals of mice used for the evaluation of in vivo therapeutic efficacy of drug-loaded liposomes in an orthotopic pancreatic tumor model. Briefly, 1 × 10^6^ AsPC-1 cells were orthotopically injected into the head of the pancreases of 6–8 weeks old female mice. After 10 days of cells implantation, mice were imaged by using IVIS and randomized into five groups (*n* = 5 in each group). Then, mice were treated with indicated groups 2×/week for 3 weeks. (**A**) Mice images showing the luciferase signals in each group. (**B**) Quantification of bioluminescence signals of respective groups. (Abbreviations: G-L represents liposomal gemcitabine; GP-L represents liposome loaded with both gemcitabine and paclitaxel; GE-L represents liposome loaded with both gemcitabine and erlotinib, GX-L represents liposome loaded with both gemcitabine and XL-184); Figure S6: endpoint bodyweight of the mice treated with indicated groups 2×/week for 3 weeks. (Abbreviations: G-L represents liposomal gemcitabine; GP-L represents liposome loaded with both gemcitabine and paclitaxel; GE-L represents liposome loaded with both gemcitabine and erlotinib, GX-L represents liposome loaded with both gemcitabine and XL-184); Figure S7: H&E staining of lung, kidney, heart, and spleen collected from mice bearing AsPC-1 xenografts after indicated treatments. (Abbreviations: G-L represents liposomal gemcitabine; GP-L represents liposome loaded with both gemcitabine and paclitaxel; GE-L represents liposome loaded with both gemcitabine and erlotinib, GX-L represents liposome loaded with both gemcitabine and XL-184); Figure S8: in vivo therapeutic efficacy of drug-loaded liposomes in an orthotopic pancreatic tumor model. Briefly, 5 × 10^3^ GFP-luciferase-labeled KPC cells were orthotopically injected into the head of the pancreases of 6–8 weeks old female C57BL/6J mice. After 10 days of cell implantation, mice were imaged by using IVIS and randomized into three groups (*n* = 5 in each group). Then, mice were treated with indicated groups 2×/week for 3 weeks. After the end of the treatment, mice were sacrificed, and endpoint tumor volume (**A**) and tumor weight (**B**) were measured. * denotes *p* < 0.05 and *** denotes *p* < 0.001 compared to vehicle, Table S1: Stability studies of liposomes: stability of liposomes estimated by measuring size and PDI of liposomes in 10 FBS containing RPMI-1640 media at 0 h, 24 h, 48 h, and 72 h, respectively, Table S2: IC-50 values respective drugs in AsPC-1 and PANC-1 cells. Cells were treated with various (mentioned in table) drug-loaded TTP-conjugated Liposomes for 72 h, Table S3: In vivo treatment doses of each drug used in our study.
