# Supplementary material for: Targeted Dual Intervention-Oriented Drug-Encapsulated (DIODE) Nanoformulations for Improved Treatment of Pancreatic Cancer
Source: Cancers (Basel). 2020 May 8;12(5):1189. doi: 10.3390/cancers12051189 (PMC7281578; doi:10.3390/cancers12051189)
Supplement: Supplementary file 1 [file cancers-12-01189-s001.pdf]

cancers

MDPI

Article

Targeted Dual Intervention-Oriented Drug-Encapsulated (DIODE) Nanoformulations for Improved Treatment of Pancreatic Cancer

Vijay Sagar Madamsetty †, Krishnendu Pal †, Shamit Kumar Dutta, Enfeng Wang and Debabrata Mukhopadhyay *

Department of Biochemistry and Molecular Biology, Mayo Clinic Florida, 4500 San Pablo Road S, Jacksonville, FL 32224, USA. madamsetty.vijay@mayo.edu (V.S.M.); pal.krishnendu@mayo.edu (K.P.); Dutta.Shamit@mayo.edu (D.S.); Wang.Enfeng@mayo.edu (E.W.)

* Correspondence: mukhopadhyay.debabrata@mayo.edu; Tel.: (904) 953-6177

† These authors contributed equally to this work.

Supplementary Figures and Figure legends:

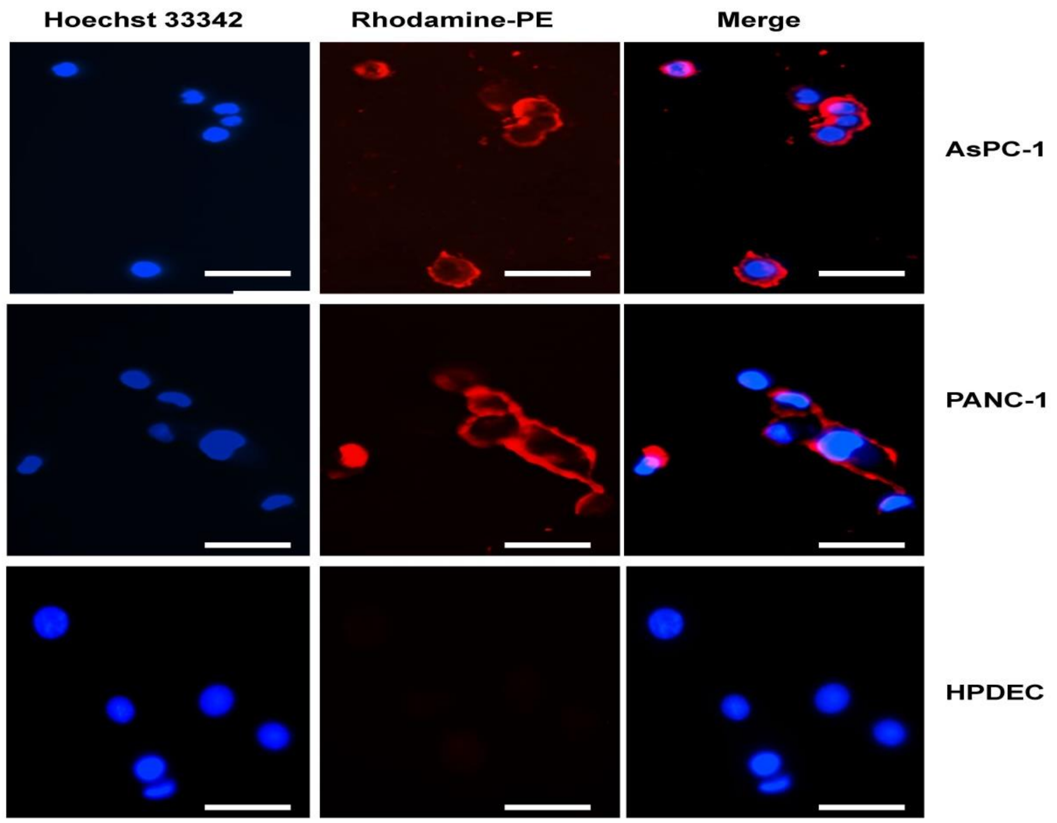

Figure S1. In vitro cellular uptake of Rhodamine-PE-labeled liposomes in pancreatic cancer cell lines. AsPC-1, PANC-1and HPDEC cells were treated with Rhodamine-PE-labeled TTP-conjugated Liposomes (TL) for four hours. Nuclei of the cells were counterstained with Hoechst 33342 for the last 30 min. Finally, cells were washed three times with PBS, and images were captured using the EVOS fluorescence microscope under the blue and red channels in 40X magnification. Bar length = 100 μm.

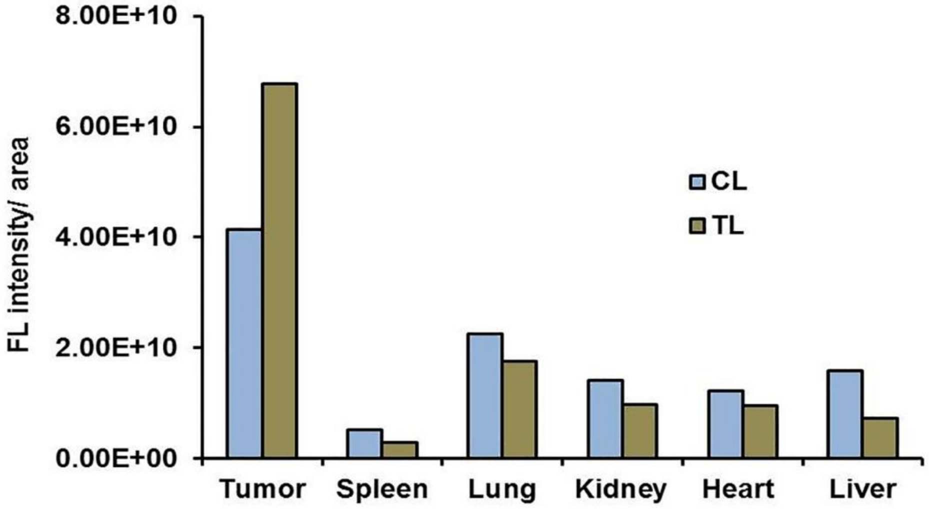

Figure S2. Quantification of fluorescence signals obtained from ex-vivo organ distribution of IR-780-dye-labeled liposomes in orthotopic pancreatic tumor bearing mice. Higher fluorescence signals were observed in tumors treated with IR-780-dye-labeled-TL-liposomes than tumors treated with IR-780-dye-labeled CL-liposomes after 48 h IV administration into mice bearing AsPC-1 orthotopic tumors in SCID mice. Abbreviations: CL, control liposome; TL, tumor targeting peptide-conjugated Liposome.

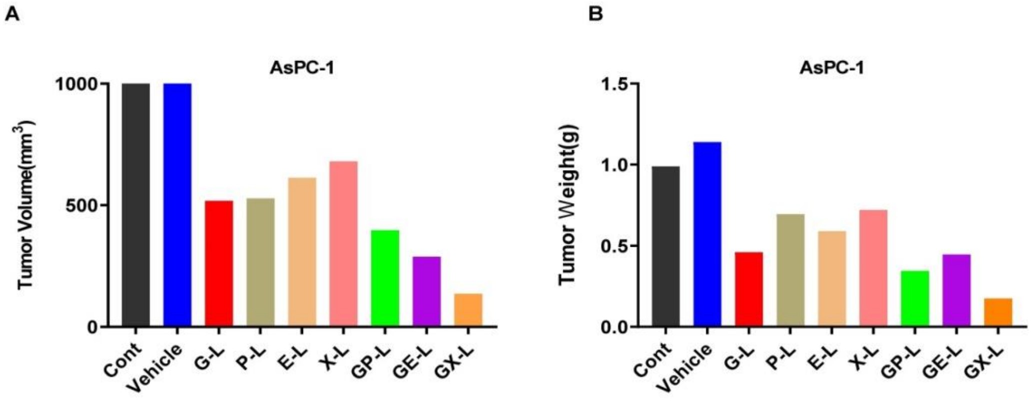

Figure S3. In vivo single mouse trial of drug-loaded liposomes in AsPC-1 xenograft. 1 × 10^6 AsPC-1 cells were orthotopically injected into the pancreas head of 8 weeks old female SCID mice. After ten days, mice were treated with drug loaded liposomes (one mouse per treatment group) 2x/wk for three weeks. Then mice were sacrificed, tumors were harvested and tumor volumes (**A**) and tumor weights (**B**) were measured. In all cases, dual-drug loaded liposomes demonstrated higher inhibition compared to single drug loaded liposomes. (Abbreviations: UT-Untreated control; L-Liposome only; G-L represents Liposomal Gemcitabine; P-L represents Liposomal Paclitaxel; E-L represents Liposomal Erlotinib, X-L represents Liposomal XL-184, GP-L represents Liposome loaded with both Gemcitabine and Paclitaxel; GE-L represents Liposome loaded with both Gemcitabine and Erlotinib, GX-L represents Liposome loaded with both Gemcitabine and XL-184).

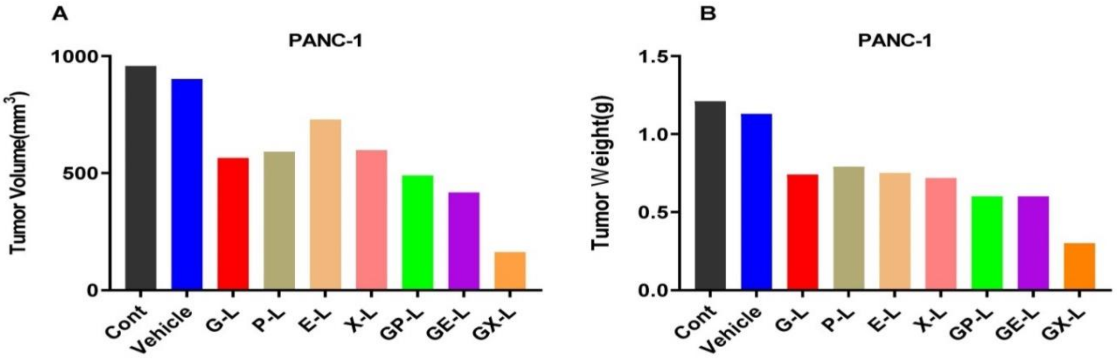

Figure S4. In vivo single mouse trial of drug-loaded liposomes in PANC-1 xenograft. 1×10^6 GFP-Luciferase labeled PANC-1 cells were orthotopically injected into the pancreas head of SCID mice. After four weeks of days, mice were treated with drug loaded liposomes (one mouse per treatment group) 2x/wk for three weeks. Then mice were sacrificed, tumors were harvested and tumor volumes (**A**) and tumor weights (**B**) were measured. In all cases, dual-drug loaded liposomes demonstrated higher inhibition compared to single drug loaded liposomes. (Abbreviations: UT-Untreated control; L-Liposome only; G-L represents Liposomal Gemcitabine; P-L represents Liposomal Paclitaxel; E-L represents Liposomal Erlotinib, X-L represents Liposomal XL-184, GP-L represents Liposome loaded with both Gemcitabine and Paclitaxel; GE-L represents Liposome loaded with both Gemcitabine and Erlotinib, GX-L represents Liposome loaded with both Gemcitabine and XL-184).

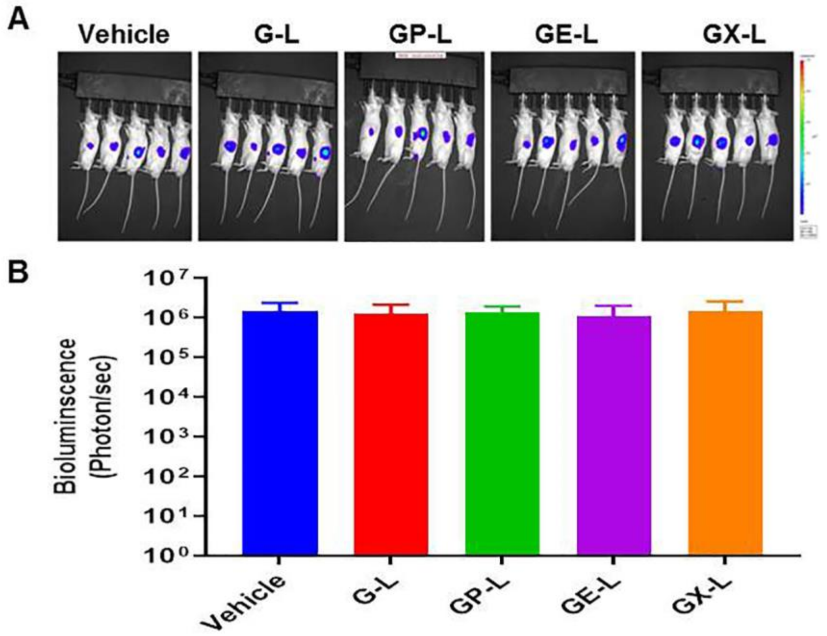

Figure S5. Initial tumor bioluminescence signals of mice used for the evaluation of in vivo therapeutic efficacy of drug loaded liposomes in an orthotopic pancreatic tumor model. 1×10^6 AsPC-1 cells were orthotopically injected into the head of the pancreases of 6–8 weeks old female mice. After ten days of cell s implantation, mice were imaged by using IVIS and randomized into five groups ($n = 5$ in each group). Then mice were treated with indicated groups 2x/wk for three weeks. (**A**) Mice images showing the luciferase signals in each group. (**B**) Quantification of bioluminescence signals of respective groups. Abbreviations: G-L represents Liposomal Gemcitabine; GP-L represents Liposome loaded with both Gemcitabine and Paclitaxel; GE-L represents Liposome loaded with both Gemcitabine and Erlotinib, GX-L represents Liposome loaded with both Gemcitabine and XL-184.

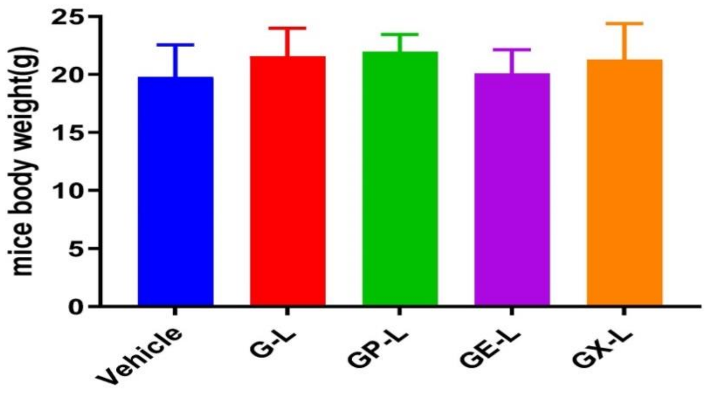

Figure S6. Endpoint bodyweight of the mice treated with indicated groups 2x/wk for three weeks.

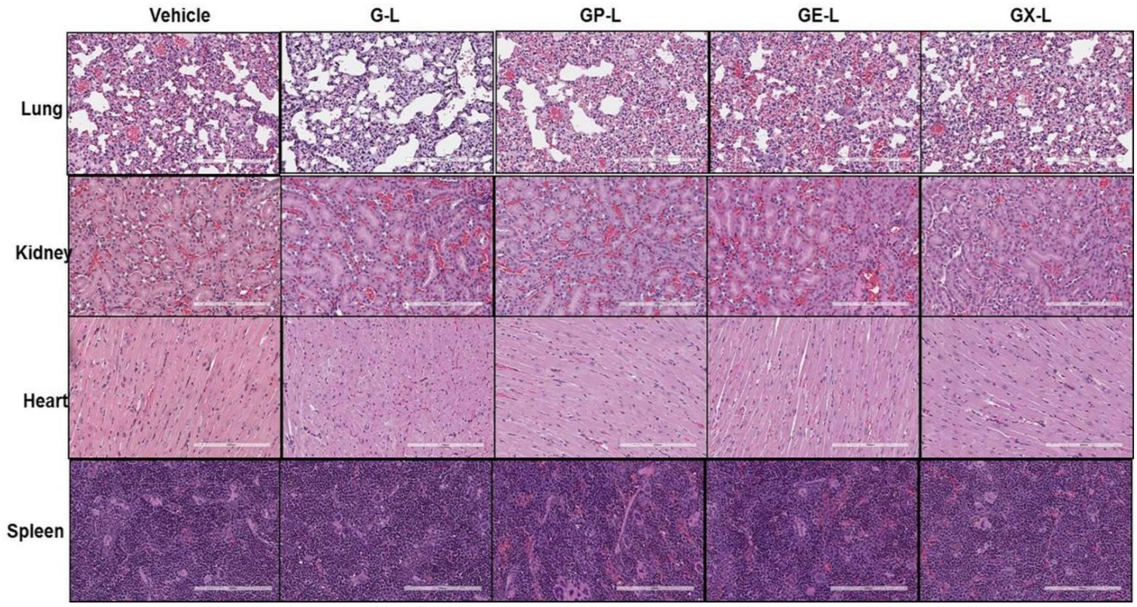

Figure S7. H&E staining of lung, kidney, heart, and spleen collected from mice bearing AsPC-1 xenografts after respective treatments. Abbreviations: G-L represents Liposomal Gemcitabine; GP-L represents Liposome loaded with both Gemcitabine and Paclitaxel; GE-L represents Liposome loaded with both Gemcitabine and Erlotinib, GX-L represents Liposome loaded with both Gemcitabine and XL-184.

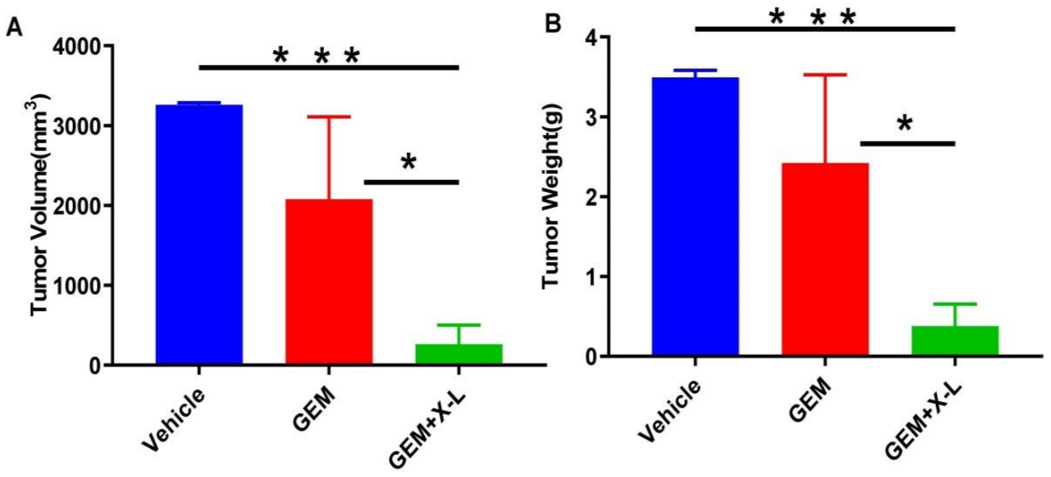

Figure S8. In vivo therapeutic efficacy of drug loaded liposomes in an orthotopic pancreatic tumor model. 5 × 10^4 GFP-Luciferase labeled KPC cells were orthotopically injected into the head of the pancreases of 6–8 weeks old female C57BL/6J mice. After ten days of cell implantation, mice were imaged by using IVIS and randomized into three groups (n = 5 in each group). Then mice were treated with indicated groups 2x/wk for three weeks. After the end of the treatment, mice were sacrificed and measure endpoint tumor volume (**A**) and tumor weight (**B**). * denotes $p < 0.05$ and *** denotes $p < 0.001$ compared to Vehicle.

Table S1. Stability studies of liposomes: stability of liposomes estimated by measuring size and PDI of liposomes in 10 FBS containing RPMI-1640 media at 0 h, 24 h, 48 h, and 72 h, respectively.

Liposome	0 h		24 h		48 h		72 h	
	**Size(nm)**	**PDI**	**Size(nm)**	**PDI**	**Size(nm)**	**PDI**	**Size(nm)**	**PDI**
L	69.4 ± 0.87	0.40 ± 0.01	60.54 ± 1.4	0.39 ± 0.01	69.48 ± 0.65	0.42 ± 0.02	55.1 ± 0.45	0.283 ± 0.004
P-L	58.7 ± 0.54	0.2 ± 0.004	58.9 ± 0.61	0.22 ± 0.01	60.76 ± 0.66	0.23 ± 0.007	62.6 ± 0.43	0.252 ± 0.006
E-L	58.3 ± 0.33	0.23 ± 0.005	58.3 ± 0.83	0.22 ± 0.001	59.25 ± 1.3	0.24 ± 0.01	60.1 ± 0.55	0.250 ± 0.003
X-L	56.0 ± 0.78	0.25 ± 0.005	56.24 ± 0.42	0.25 ± 0.007	56.78 ± 0.35	0.26 ± 0.004	89.9 ± 2.5	0.485 ± 0.085
G-L	57.1 ± 0.18	0.21 ± 0.004	57.32 ± 0.58	0.23 ± 0.002	58.93 ± 0.5	0.24 ± 0.002	61.9 ± 1.08	0.255 ± 0.002
GP-L	57.8 ± 0.53	0.22 ± 0.01	58.31 ± 0.22	0.22 ± 0.01	58.51 ± 0.43	0.23 ± 0.005	59.3 ± 0.51	0.238 ± 0.008
GE-L	57.1 ± 0.5	0.22 ± 0.004	57.47 ± 0.36	0.22 ± 0.006	57.23 ± 0.48	0.23 ± 0.01	58.1 ± 0.30	0.234 ± 0.005
GX-L	58.1±0.2	0.21 ± 0.004	59.2 ± 0.54	0.23 ± 0.006	59.98 ± 1.09	0.23 ± 0.009	62.5 ± 0.43	0.245 ± 0.011

Abbreviations: L-Liposome only; P-L represents Liposomal Paclitaxel; E-L represents Liposomal Erlotinib, X-L represents Liposomal XL-184; G-L represents Liposomal Gemcitabine; GP-L represents Liposome loaded with both Gemcitabine and Paclitaxel; GE-L represents Liposome loaded with both Gemcitabine and Erlotinib, GX-L represents Liposome loaded with both Gemcitabine and XL-184.

Table S2. IC-50 values respective drugs in AsPC-1 and PANC-1 cells. Cells were treated with various (mentioned in table) drug-loaded TTP-conjugated Liposomes for 72 h.

**Liposome**	**IC-50 of drugs (μM)**	
	**AsPC-1**	**PANC-1**
**P-L**	2.4	1.868
**E-L**	6.37	5.25
**X-L**	4.20	3.413
**G-L**	3.289	1.33
**GP-L**	1.94 (G) ; 0.1926 (P)	0.671 (G); 0.066 (P)
**GE-L**	2.04 (G) ; 1.25 (E)	0.74 (G) ; 0.45 (E)
**GX-L**	1.99 (G) ; 0.355 (X)	0.984 (G); 0.175 (X)

Abbreviations: P-L represents Liposomal Paclitaxel; E-L represents Liposomal Erlotinib, X-L represents Liposomal XL-184; G-L represents Liposomal Gemcitabine; GP-L represents Liposome loaded with both

Gemcitabine and Paclitaxel; GE-L represents Liposome loaded with both Gemcitabine and Erlotinib, GX-L represents Liposome loaded with both Gemcitabine and XL-184.

Table S3. In vivo treatment doses of each drug used in our study.

**Liposome**	**The dose of each drug used in our studies (mg/kg)**
P-L	**0.405**
E-L	**0.707**
X-L	**0.495**
G-L	**0.905**
GP-L	**0.90 (G) ; 0.288 (P)**
GE-L	**0.845 (G); 0.77 (E)**
GX-L	**0.90 (G); 0.37856 (X)**

Abbreviations: P-L represents Liposomal Paclitaxel; E-L represents Liposomal Erlotinib, X-L represents Liposomal XL-184; G-L represents Liposomal Gemcitabine; GP-L represents Liposome loaded with both Gemcitabine and Paclitaxel; GE-L represents Liposome loaded with both Gemcitabine and Erlotinib, GX-L represents Liposome loaded with both Gemcitabine and XL-184.
